# Special Issue “Personal Therapy for Blood Disorders”

**DOI:** 10.3390/jpm13020339

**Published:** 2023-02-15

**Authors:** Maria Hernandez-Valladares

**Affiliations:** 1 Institute of Biotechnology, University of Granada, Avenida de la Fuente Nueva S/N, 18071 Granada, Spain; mariahv@ugr.es; Tel.: +34-958241000 (ext. 20284); 2 Instituto de Investigación Biosanitaria ibs.GRANADA, 18012 Granada, Spain; 3 Department of Physical Chemistry, University of Granada, Avenida de la Fuente Nueva S/N, 18071 Granada, Spain

This editorial of the Special Issue “Personal Therapy for Blood disorders” aims to draw more attention to blood cancer heterogeneity and personalized strategies for diagnosis, prognosis and therapeutic treatment. Personalized approaches can provide more accurate diagnosis and therapies than standard procedures established from large population studies or by cohort-based decisions. This Special Issue invites research groups to share their most recent findings, especially from molecular profiles obtained using the different omics technologies, toward approaches for individualized treatments. The debate on the implementation of personalized molecular approaches in the clinical setting and how patients could be integrated in the decision-making process is especially welcome.

**Recognition of the molecular heterogeneity as the cornerstone of efficient anti-leukemia therapies.** Acute lymphoblastic leukemia is the most common childhood cancer and it arises in hematopoietic stem cells, which change into malignant white blood cells through the accumulation of genetic mutations [1]. No cells are genetically identical and numerous genetic mutations are usually present at diagnosis. Acute myeloid leukemia (AML) is the most common form of acute leukemia in adults and accounts for the highest percentage of leukemia death [2]. It is found that more than 95% of AML patients have driving and co-concurring mutations regardless of the presence of cytogenetic abnormalities [3]. Therefore, understanding the heterogeneity of leukemia is relevant to further improve current treatment options and develop new multi-targeted therapies. Obviously, this genomic heterogeneity is reflected at the proteome and metabolome level. Human proteomes are very complex. Allelic variations, alternative splicing of RNA transcripts and many post-translational modifications contribute to the existence of several hundred thousand proteoforms [4]. Regarded together, it seems that the characterization of the multiple “omes” becomes crucial to understanding the complexity of the disease and ensuring effective therapeutic decisions.

**All-patients omics versus stratified omics analyses**. Liquid chromatography-mass spectrometry (LC-MS) analysis of proteomes and metabolomes provides global or targeted identifications and quantitative values of the proteins and metabolites of the study subjects [5,6]. In the past, LC-MS-based omics studies have used leukemic cell lines (low heterogeneity samples) and human cohorts (high heterogeneity samples) comprising patient samples of diverse cytogenetic and genomic backgrounds. Proteomics and phosphoproteomics studies in primary cells from 41 AML patients with diverse cytogenetic and mutational profiles associated relapses with increased an expression of RNA-processing proteins and with an increase in phosphorylation events by cyclin-dependent kinases (CDKs) and casein kinase 2 (CSK2) [7]. Lately, accounting for the leukemic heterogeneity, several publications have reported proteomic and metabolomic profiles of patient groups with specific cytogenetics and carrying the same gene variations. This has resulted, as an example, in the identification of the Wee1-like protein kinase (WEE1)-cyclin-dependent kinase 1 (CDK1) axis in the resistance of tyrosine kinase inhibitors therapy in receptor-type tyrosine-protein kinase FLT3-internal tandem duplications (FLT3-ITD)-positive AML patients [8]. This study suggested a combined therapy of WEE1 and tyrosine kinase inhibitors to improve patients’ clinical outcomes. However, a recent proteogenomic study has shown that genomic aberrations do not exclusively determine AML proteomic subtypes and only one subtype, the Mito-AML captured at the proteome level, is characterized by a high expression of mitochondrial proteins and confers poor outcome [9]. 

Considering everything, the leukemia heterogeneity does not allow for categorizing all the patients based on their cytogenetics and genomic profiles. Therefore, omics strategies that aim to "molecularly" describe patients at different levels should be utilized to decide the best therapeutical approach. 

**Implementation of the omics technologies in clinical settings.** The above reflections along with the current original research and literature reviews highlight the need to investigate individual molecular profiles to benefit from targeted therapies and decrease the deleterious effects of untargeted strategies [10]. This Special Issue aims to improve our understanding of leukemia heterogeneity and its effects, and how we can take advantage of current omics technologies to narrow down the selection of key therapeutic targets for each patient. Although sample preparation and data analysis protocols could be implemented in the clinic, it is not clear yet how the different omics applications can be carried out and who would oversee the performance and result delivery of these technologies. Although clinicians are welcome in research activities, initiatives that support investigators to work in clinical settings might alleviate the transition from laboratory-produced omics to hospital-produced omics characterizations.
